# Custom-made reverse shoulder arthroplasty for severe glenoid bone loss: review of the literature and our preliminary results

**DOI:** 10.1186/s10195-020-00564-6

**Published:** 2021-01-19

**Authors:** G. Porcellini, G. M. Micheloni, L. Tarallo, P. Paladini, G. Merolla, F. Catani

**Affiliations:** 1 Department of Orthopaedic Surgery, University of Modena and Reggio Emilia, Modena, Italy; 2 Unit of Shoulder and Elbow Surgery, Ospedale Cervesi, Cattolica, RN Italy

**Keywords:** Severe bone loss, Custom-made implant, Reverse shoulder arthroplasty, Bone stock

## Abstract

The treatment of severe glenoid bone loss in shoulder arthroplasty represents a challenge, and the results of current prosthetic designs with only glenoid fixation still remain unsatisfactory. In the past decade, customized glenoid prostheses have been developed to address severe glenoid arthritis and in the revision setting. In this review, we analyzed the current surgical options, the classification limits, past literature evidence, and our preliminary results of 6 patients (3 male, 3 female) treated with a reverse implant and custom-made glenoid implant (ProMade; LimaCorporate, Italy). Computer analysis of the residual shape and the amount of glenoid bone stock in association with new classifications could help the surgeon to obtain good clinical and radiological outcomes. The development of navigation systems could improve the adequacy of the implant and, thus, the reliability and longevity of the implant itself.

## Introduction

The management of glenoid bone loss is a highly demanding challenge for orthopedic surgeons, and the number of cases is expected to rise in the future, in proportion to the increase of prosthetic implants, life expectancy, and functional demand of patients [1–3].

The main causes of bone defects include degenerative bone diseases of the glenohumeral joint, chronic dislocations, congenital malformations, sequelae of autoimmune diseases including rheumatoid arthritis, status post proximal humerus fractures treated with fixation devices (intramedullary plates or nails) secondary to damaging of the glenoid articular surface due to extrusion or migration of fixation devices [4]. Further recurrent reasons are related to previous prosthetic implant complications—septic or aseptic mobilization of the glenoid component in anatomical or reverse shoulder implants frequently induces a significant loss of glenoid bone stock, forcing the surgeon to make extremely difficult decisions in order to guarantee the best compromise between functionality and pain regression.

Glenoid bone loss can be mild (B2 and B3 according to Walch classification) and is usually treated with eccentric glenoid reaming, hemiarthroplasty, bone grafts, and augmented implants [5–8]; or it could present significant concentric or eccentric defects [6, 9].

The definition and quantification of severe bone loss of the vault and the glenoid surface are controversial. In our experience, we consider a case to be ‘severe’ if it is impossible to treat with traditional implants including the use of wedges.

## Classifications

Various classifications are present in the literature, but the most commonly used are the classifications proposed by Antuna and Seebauer.

Antuna et al. classified glenoid bone loss as central, peripheral, and combined, with each group being partitioned into mild, moderate and severe [10].

Seebauer et al. classified the defects as centric and eccentric erosions. Centric defects (contained) are shallow (C1), deep (C2), cavitary (C3) or destructive (C4), whereas eccentric defects are further partitioned based on size and location [4].

The above-mentioned classifications are particularly useful to describe the defects, although less beneficial in terms of treatment and surgical plan.

In severe combined defects (according to Antuna classification), or in C4 and E4 defects (according to Seebauer classification), the decision-making process is challenging.

Identifying a new classification system for severe bone loss based on a three-dimensional (3D) reconstruction computed tomography (CT) scan (according to Seebauer classification), residual bone quality and shoulder stability could be useful to obtain common, shareable, and reliable surgical treatment solutions. The aim of this classification is to find cases that are potentially treatable with traditional implants and cases requiring custom-made implants.

## Surgical treatments

Glenoid bone loss can be treated with reverse shoulder arthroplasty (RSA) and bone grafting. These techniques allow the management of mild-to moderate defects with autologous bone or titanium wedges/hemi-wedges placed on the basis of bone loss morphology and extension.

Boileau et al. proposed the bony increased offset (BIO) technique, with compression of the bone graft by the implant to achieve a more favorable environment for graft incorporation. The main goal of this technique is to achieve lateralization of the glenohumeral center of rotation. The size and morphology of the bone graft are determined by the extent of the bone defect, the joint line, and soft-tissue tensioning [11]. Boileau et al. reported encouraging outcomes with a graft incorporation rate of 98% in a study of 42 patients treated with BIO-RSA, although the major criticism was related to the risk of graft non-union and graft reabsorption [12]. In our opinion, the management of glenoid retroversion could become challenging with this surgical solution. Additionally, eccentric bone grafts show a lower union rate compared with concentric grafts as recently reported in the literature [13].

Other authors have suggested augmented glenoid implants to restore the anatomic joint line and minimize the non-union risk [14]. Studies on early metal-backed wedge-shaped glenoid augments in anatomic implants reported a high failure rate after a 10-year follow-up evaluation [8, 15]. Recently, the introduction of trabecular metal components has renewed the interest in these types of implants with excellent early outcomes [16].

The major difficulties are in the management and treatment of severe glenoid bone loss, which are unmanageable in cases of the above-listed prosthetic implants. Some authors suggest the use of endoprosthesis, although various studies in the literature have shown highly scarce and mediocre clinical outcomes when compared with the use of total shoulder replacement [17–22]. Other authors have suggested the use of bone grafting, although there is also insufficient evidence of clinical outcomes and many critical aspects to report, i.e., the demanding surgical technique and the potential risk of bone reabsorption over time and, hence, mobilization of the implant itself [7, 18, 23–29].

## Custom made-implants

Indications for custom-made reverse implants are related to the patient’s functional demand and to the remaining bone stock. Custom-made implants are not recommended in patients with scarce or reduced functional demand, considering the elevated costs and not yet standardized results.

A flat glenoid metal back, in the case of severe bone stock, has an extremely low sitting percentage and the central peg does not ensure adequate stability.

A minimum vault depth of 10 mm to achieve initial fixation and a volume that allows the insertion of a minimum of two screws is reported in the literature [30]. Furthermore, there is the consensus that implant stability is achieved if the peg is almost 50% in length into the glenoid bone [31].

The use of 3D reconstruction CT scans, with specific metal artifact reduction (MAR) software, associated with the development of custom-made implants in orthopedic surgery, provides interesting solutions for cases of severe glenoid bone loss.

Progress in 3D printing technology has led to the manufacture of an implant that matches, as accurately as possible, the glenoid deformity [32].

Patient-specific instrumentation (PSI) improves the accuracy of component implantation as planned in the preoperative analysis.

**Fig. 1:**
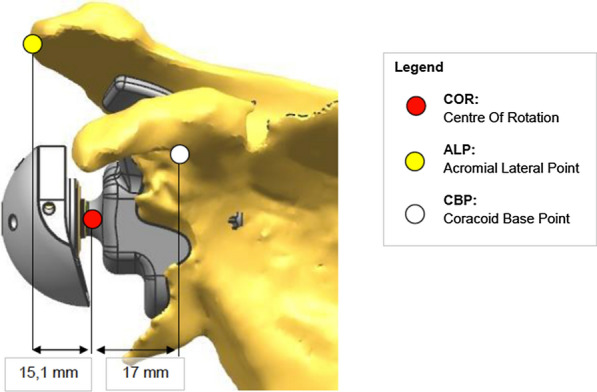
Center of rotation and landmarks for joint line reconstruction

The advantages of custom-made surgery include the possibility to determine the stability on elements that are still intact (i.e., the spine of the scapula or the coracoid process), to fill the gap in the bone and to adequately reconstruct the original joint line, to place the fixation devices (screws) with the proper length, and to determine the best orientation (Fig. 1). This technology is based on preoperative CT scans and analyzed by computerized systems able to identify the bone loss entity and to determine the fixation elements in which the glenoid component of the implant is to be placed.

## Preoperative Assessment and Computer-Assisted Design/Computer-Assisted Manufacturing (CAD/CAM) analysis

Anatomical reconstruction, and evaluation of the patient’s image data congruency and quality are performed during the initial phase (i.e., congruence of anatomical site, opportunity to locate and define anatomical landmarks, slice thickness, presence, and entity of metal artifacts, ‘loose’ bone areas, areas to be removed or to be preserved), consistently with the selected surgical technique and implant insertion direction.

Anatomical reconstruction is performed using commercially available Materialize Mimics Suite software.

It is essential that the evaluation is performed on the different areas of the scapula and humerus, including determining the impact of the above-mentioned parameters on the geometrical precision, the possible design, and the surgical technique.

In each case, the design is directed to optimize the metal–bone interface area, the primary stability, and the load transfer, with metal engagement on the most supportive areas, with an ultimately consecutive phase of bone remodeling (drilling, curettage, reaming, high-speed burr).

In revision cases, it is frequently essential to compromise on bone sparing and bone removal in the surgical technique, since it is commonly related both to metal artifacts and to uncertainty of bone loss because of the hardware removal phase.

A wrong estimation of the bone loss/bone not visible (due to artifacts or revision procedure) could lead to a loose custom implant or an extremely tight-fitting implant and manual preparation would therefore be required.

Thus, in order to overcome this situation, planning an adequate guided drilling/reaming and determining the reliable landmarks and the stable bone areas could allow a proper fitting in the above-mentioned cases.

The design of the implant will have to allow primary stability, recovery of biomechanical balance, and adequate range of motion.

A 3D reconstruction facilitates specific planning of the features of the implantable component, in addition to patient-specific instruments and surgical technique, essential for cautious and guided preparation of the site and to identify the anatomical landmarks on the patient’s anatomy and on anatomical replicas.

Frequently, custom glenoid implants are used when a standard implant would not allow adequate stability or stress distribution. Hence, to overcome the limitation of conventional implants, customization facilitates an increase of the fixation site, progressively growing implant invasiveness toward the coracoid process, increasing peg dimension or length, uncoupling peg direction with glenosphere coupling direction, adding localized screws or, in other cases, stabilizing the system with acromial support pads and flanges.

The best production process is evaluated in order to optimize the material shape and performance during the design phase. For instance, maximizing the use of high integration surfaces in contact with native bone (in the case of soft-tissue reattachment sites although limiting the presence of abrasive surfaces or cutting edges in which soft-tissue sliding over implant is noted), maximizing flexible structures that could permit to fill the gaps or transfer loads from the implant to the bone effortlessly, allowing the required resistance to the supportive structures.

Design and CAM preparation are prepared by means of the CAD system (Siemens NX Suite, solid modeling and CAM) in order to allow consistent and precise preparation of the 3D models for additive manufacturing and standard production processes, where precision is required. The use of non-technical 3D visualization and interaction tools are fundamental in order to permit the surgeon to evaluate the 3D reconstruction and the anatomy/implant/instrument interaction, essential for the challenging analysis of highly deformed anatomies, frequently associated with custom cases.

## Preliminary experience and results

Six patients (3 male, 3 female) treated with a reverse implant and custom-made glenoid implant (ProMade; LimaCorporate, Italy) at the Ospedale Cervesi of Cattolica (Rimini, Italy) and at the Policlinico of Modena (Modena, Italy) were included and evaluated in our case study.

The mean age of the patients at the time of the surgical procedure was 64 years (minimum 48 and maximum 74 years) and the mean follow-up time was 31.67 months (minimum 25 and maximum 38 months). A case of painful partial replacement in status post previous surgical procedures, a case of an infected anatomical implant, a case of status post scapular fracture in a patient with rheumatoid arthritis, a case of status post fracture treated with the open reduction internal fixation technique, two cases of mobilization of reverse implant in status post previous surgical procedures.

According to Antuna classification, each case presented severe combined bone loss and according to Seebauer classification, four cases were E4 and two cases were C4.

**Fig. 2:**
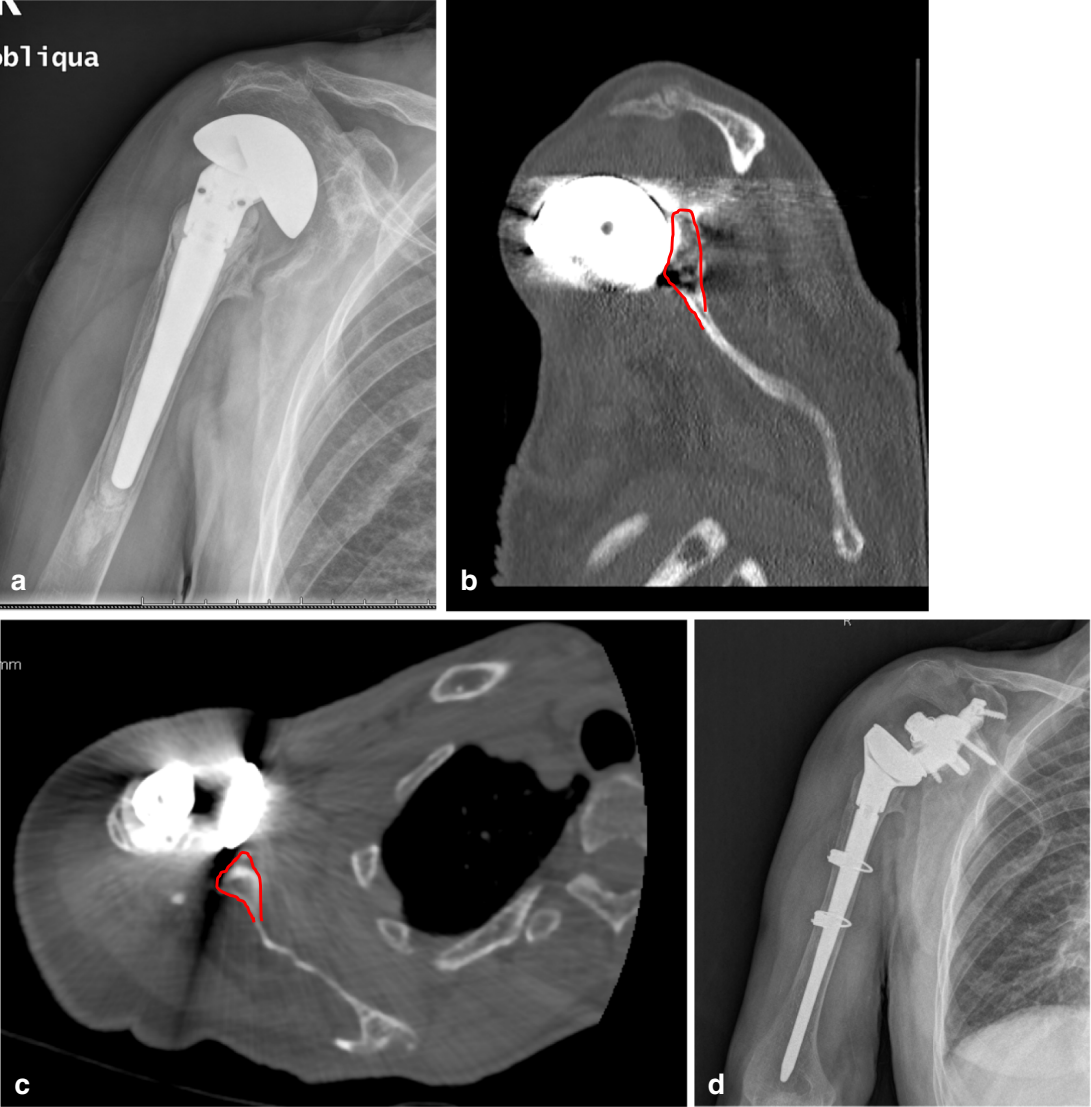
**a** Preoperative X-rays, **b** preoperative sagittal plane CT scan, **c **preoperative oblique plane CT scan, **d** postoperative X-rays

Each of our patients underwent preoperative clinical evaluation (Constant score, ASES score, VAS scale), radiological assessment and 3D CT scan with specific MAR software (Fig. 2a–d).

The CT scan for each case was presented to LimaCorporate in order to analyze the size and shape and to select the most adequate fixation elements to obtain stability of the glenoid component.

The LimaCorporate Company provided the orthopedic surgeon with a file containing images, information, an accurate description of the entire surgical steps to be followed, and any related critical issues.

The utilized material was entirely PSI (cutters, trays, and screws), obtained with CAD and CAM reconstructions, including the 3D model to simulate the definitive glenoid component for the best orientation.

In each case, samples of tissue were collected intraoperatively and sent to the laboratory for prompt histologic examination.

All patients underwent postoperative radiographs and serial clinical and radiological follow-up evaluations after being discharged.

The Constant score, the ASES score and the pain VAS scale were used to determine the clinical outcomes. We observed an increased range of motion regarding the anterior and lateral elevation (mean increase 10.00 ± 23.45 and 10.00 ± 25.39, respectively). The difference between the internal and the external rotations showed no significant results statistically.

The mean pain reduction was 5.67 ± 1.63 according to the pain VAS scale. One patient complained of persistent pain at the end of the follow-up time.

The mean increase in the Constant score and the ASES score was 9.83 ± 5.60 and of 30.57 ± 10.77, respectively (Table 1).

**Table 1:** Summary of patient characteristics

Name	Gender	Age	Classification (Antuna)	Classification (Seebauer)	CS preop	CS postop	ASES preop	ASES postop	VAS preop	VAS postop
CM	F	74	Severe combined	E4	5	12	10	25	8	5
OF	F	66	Severe combined	C4	13	17	25	55	6	0
VG	M	71	Severe combined	E4	18	38	23.3	71.6	8	0
DBN	M	48	Severe combined	E4	20	28	13.3	40	8	2
BN	F	56	Severe combined	E4	18	26	16.7	46.7	8	3
MW	M	69	Severe combined	E4	16	28	3.3	36.7	10	4

Name	Ant. elev. preop	Ant. elev. postop	Abduction pre	Abduction post	Intrarotation pre	Intrarotation post	Extrarotation pre	Extrarotation post	Notching (y/n)
CM	15°	25°	15°	25°	Lateral thigh	Lateral thigh	< 10°	< 10°	y
OF	35°	NA	40°	NA	Lateral thigh	Lateral thigh	< 10°	NA	n
VG	70°	90°	60°	80°	Gluteus	Gluteus	< 10°	< 10°	n
DBN	60°	70°	30°	50°	Lateral thigh	Lateral thigh	< 10°	< 10°	n
BN	40°	65°	30°	50°	Lateral thigh	Lateral thigh	< 10°	< 10°	n
MW	30°	60°	40°	70°	Lateral thigh	Lateral thigh	< 10°	< 10°	n

*NA* not applicable, *y* yes, *n* no

The evaluation of radiolucent lines, signs of mobilization and potential glenoid notching was based on the radiological findings. None of the patients presented glenoid notching or implant mobilization. Two cases showed evidence of radiolucent lines <2 mm, which did not lead to changes or worsening of the clinical scores. No progression of such lines was evident until the end of the follow-up phase.

One female patient presented an episode of non-traumatic partial dislocation of the implant during the postoperative first trimester. The complete resolution of the correct implant stability was obtained with athletic taping and a physical therapy plan focused on rehabilitation and recovery of the correct shoulder mobility pattern.

## Discussion and review of the literature

The limited number and the heterogeneity of the patients unquestionably complicate our observations and the preoperative studies based on the CT scan imaging (that guarantees a good resolution of shapes and planes) would not be sufficient to determine the areas with the best bone density. Furthermore, the time between the CT scan and the surgical procedure, approximately 3 months, could lead to additional bone and soft-tissue changes.

During the surgical procedures, the major difficulty we faced in all patients was the correct preparation of the portion of the remaining glenoid component in order to place the implant. We encountered anatomical changes, scar, and fibrous tissues because of the status post previous (often multiple) surgical procedure. Additionally, the placement of cut guides and trial components required a high degree of surgical expertise and perfectly accurate movements. The variation of a few grades when placing the PSI components, considering the presence of soft tissues not visualized with the CT scan, could cause a significant change in the orientation of the definitive component. Similar observations include the anchoring screws of the glenoid component; perforating in different directions compared to the planned direction might lead to positioning them in areas that do not guarantee correct primary stability of the implant.

**Fig. 3:**
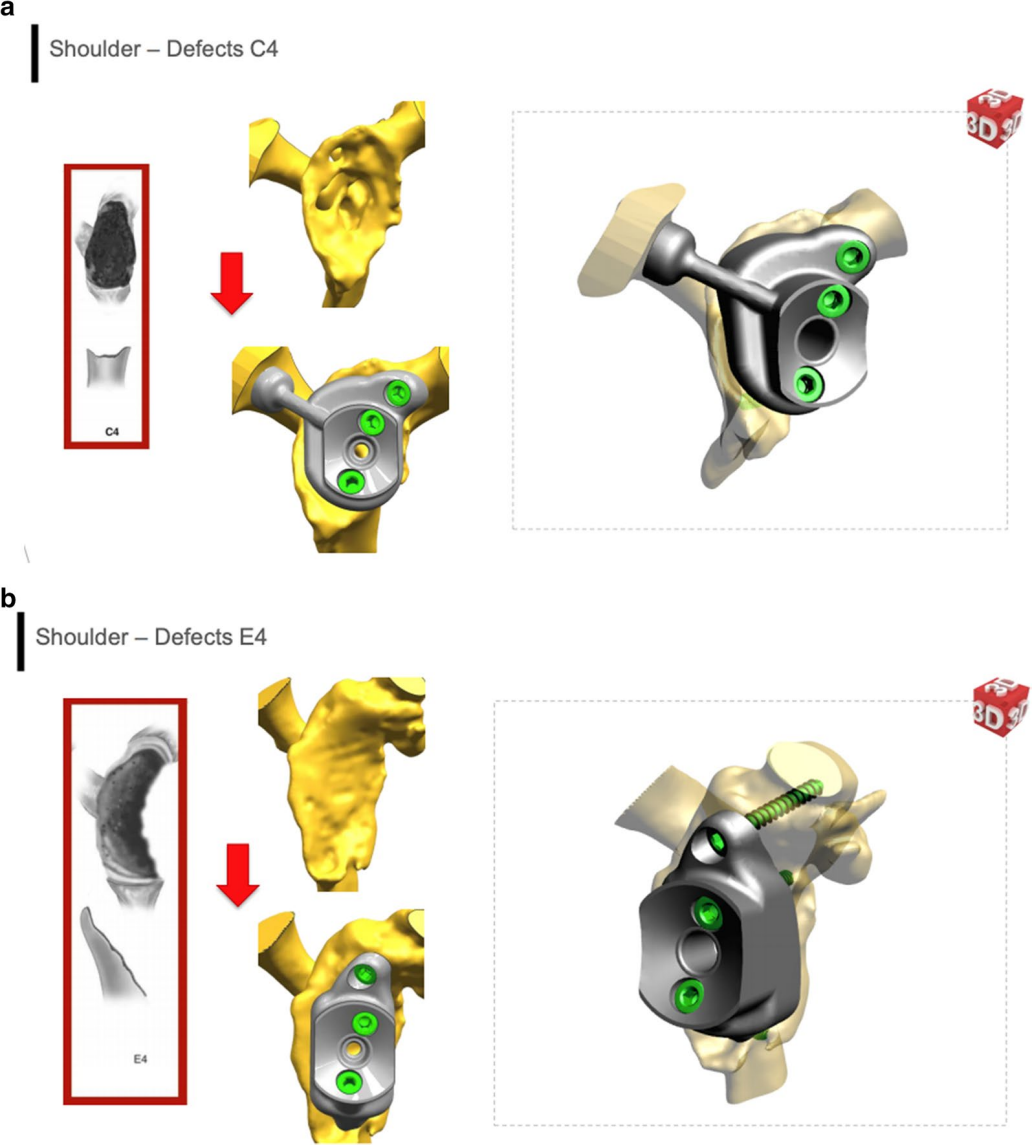
**a** Defect C4 according to Seebauer classification and custom implant with coracoid fixation. **b** Defect E4 according to Seebauer classification and custom implant with posterior wedge

The development of computer-aided surgery technologies and intraoperative navigation, recently introduced in shoulder replacement surgery, could probably be significantly useful and could overcome such intraoperative difficulties. Image-guided surgery and intraoperative feedback of the preoperative data and parameters are mandatory, particularly in difficult cases where accurate implant positioning is essential to obtain satisfactory results. Another focal point will be establishing a classification that allows the surgeon to select the correct custom implant based on bone loss morphology and extension. Eccentric and concentric defects require different solutions to obtain proper stability and fixation (Fig. 3a, b).

There are few studies in the literature regarding clinical experience with custom-made devices, and the outcomes, although encouraging, present a greatly reduced follow-up time.

A case report by Stoffelen et al. [32] concerning treatment with a glenoid custom-made anatomical total shoulder replacement, reported abduction of 90°, elevation of 110°, external rotation of 40° and internal rotation at T12, with a Constant score of 51 at 2.5 years of follow-up.

A study by Chammaa et al. [1] in 37 patients treated with custom-made implants (CAD–CAM total shoulder replacement) for severe glenoid bone loss, obtained a postoperative pain level of 2.4. The mean active anterior elevation ranged from 39° to 63° and the external rotation from 6° to 15°. During the 5-year follow-up period, 6 of the 37 patients underwent an additional surgical procedure due to complications such as aseptic mobilization, fractures, and dislocation of the implant.

Dines et al. reported the case of a patient treated with the patient-specific Glenoid Vault Reconstruction System developed by the authors in conjunction with Comprehensive Shoulder Arthroplasty System (Zimmer Biomet). After 18 months, the active range of motion of elevation forward was 130° and 20° for external rotation [33].

In a multicenter study by Debeer et al. of 10 patients treated with the Glenius Glenoid Reconstruction System, the mean patient-derived Constant-Murley score was 41.3 ± 17.5 points (range 18–76 points) with a VAS scale of 3.3 ± 2.5 points (range 0–7 points) at 30.5 months of follow-up [34].

In our study, we observed a slight improvement of the anterior and lateral elevation, possibly related to restoration of the center of rotation and to lateralization of the glenohumeral rotation center. This could have optimized the role of the deltoid for elevation of the limb, although with no improvement on the external rotation. The lack of internal rotation improvement could be related to degeneration of the subscapularis, further weakened by a new surgical access.

As well as the moderate increased range of motion, the patients were extremely satisfied with the reduction in perceived pain, a fundamental feature of the modest quality of life of patients before surgical treatment. Only one patient reported pain at the end of the follow-up period, although gradually reducing.

The dislocation reported in the postoperative period could be attributed to weakness of the anterior portion of the deltoid, in relation to the previous multiple surgical procedures. The recovery of stability consequent to the physical therapy appears to confirm this hypothesis.

The follow-up period is relatively brief and it is essential to monitor the above-mentioned changes in the future and possible evolution over time.

The radiological results were also very encouraging—the onset of radiolucent lines noted in two cases did not reflect pain-related symptoms, and did not negatively interfere with the clinical outcomes.

**Fig. 4:**
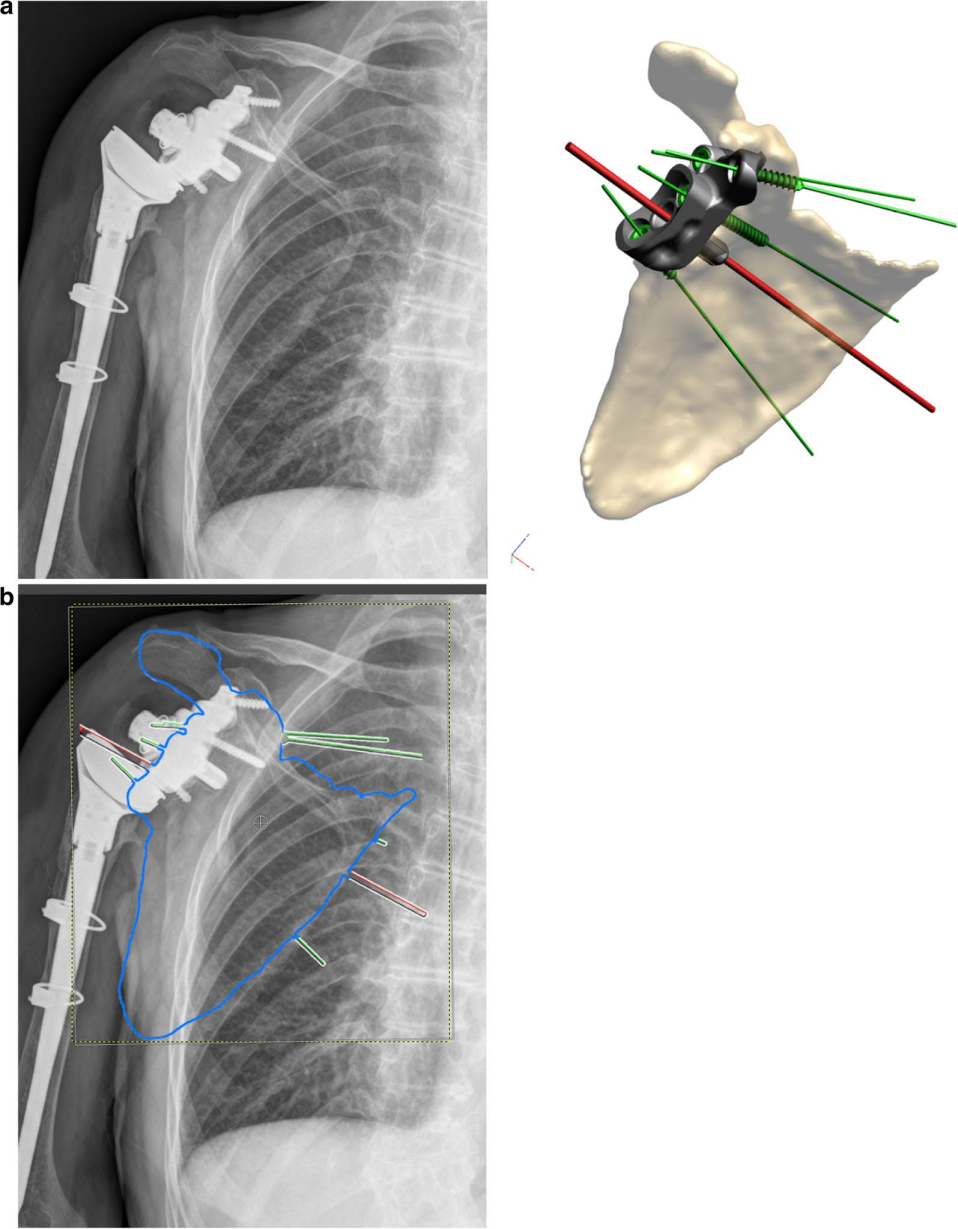
**a** Comparison between postoperative X-rays and preoperative plan. **b** Matching between X-rays and preoperative plan with reconstruction of peg and screw directions

In fact, it is difficult to compare the planned images with the postoperative radiographs, although the directions of peg and screws are detectable (Fig. 4a, b).

Furthermore, the elevated costs related to the custom-made implant surgery require observation. The production of custom-made components and the work of the technicians unquestionably present certain advantages, although this technology is not applicable on a large scale.

This study does not aim to analyze the cost/benefits, and additional studies regarding this specific evaluation are required. Nevertheless, we can confirm that extremely accurate patient selection based on a patient’s functional demand, pain-related symptoms, and motivation is required at the present time.

## Conclusions

In our experience, the use of glenoid custom-made components based on CT scans results has been shown to be a potential solution for the management of severe glenoid bone loss, with encouraging clinical and radiological outcomes, although with a limited follow-up time.

These implants should be performed by dedicated surgeons with a high degree of experience in shoulder prosthesis, who are able to overcome unavoidable intraoperative difficulties.

The most reliable outcome of custom-made implants in severe glenoid bone loss is the decrease in pain. A large variety of outcomes were noted regarding functionality and, therefore, a significant clinical improvement is not guaranteed. Computer analysis of residual shape and amount of glenoid bone stock, in association with new classifications, could enable the surgeon to obtain good clinical and radiological outcomes, including the most difficult cases.

## Data Availability

All data generated or analyzed during this study are included in this published article.
